# A scoping review of overweight and obesity prevalence and determinants among immigrant adults in Nordic countries

**DOI:** 10.1016/j.jmh.2025.100377

**Published:** 2025-11-01

**Authors:** Luna Emilia Kronstad, Miroslava Tokovska, Adnan Kisa

**Affiliations:** a School of Health Sciences, Kristiania University of Applied Sciences, Oslo, Norway; b School of Health Sciences, Kristiania University of Applied Sciences, Prinsens Gate 7-9, 0152, Oslo, Norway

**Keywords:** Obesity, Overweight, Immigrant adults, Nordic countries, Risk factors, Health interventions

## Abstract

**Background:**

Overweight and obesity are major public health challenges worldwide and are disproportionately prevalent among immigrant populations in Western countries, including the Nordic region. However, evidence on their determinants and effective prevention strategies within Nordic immigrant populations remains fragmented.

**Purpose:**

This study aims to map existing research on the prevalence, determinants, and interventions related to overweight and obesity among adult immigrants in Nordic countries.

**Method:**

A scoping review of peer-reviewed studies published between 2013 and 2023 was conducted using CINAHL, PsycINFO, PubMed, and Web of Science to systematically synthesize evidence on these themes.

**Results:**

Immigrants generally exhibited higher rates of overweight and obesity compared with native-born populations, with specific groups such as Somali and Iraqi immigrant women experiencing notably elevated prevalence. Key determinants included low physical activity, high-energy diets, socioeconomic disadvantage, acculturation challenges, and prolonged residence in the host country. Interventions included culturally adapted prevention programs promoting healthier diets, increased physical activity, and lifestyle modification. Promising approaches included gender-specific groups led by culturally sensitive health coaches, cooking classes with motivational support, and community-based physical activity initiatives.

**Conclusion:**

Addressing obesity among immigrant populations in the Nordic region requires culturally responsive, community-engaged, and evidence-informed public health strategies. This review emphasizes the importance of integrating cultural competence, gender sensitivity, and structural supports to promote healthy lifestyles and advance equity in obesity prevention.

## Highlights

• Immigrant adults in the Nordic countries have higher rates of overweight and obesity than native populations, with Somali and Iraqi women showing the highest prevalence.
• Key risk factors include low physical activity, high-energy diets, socioeconomic disadvantage, limited language proficiency, acculturation challenges, and long duration of residence.
• Traditional gender roles, social norms around food and hospitality, and restricted access to gender-appropriate exercise settings elevate risk, especially among women.
• Programs featuring culturally and linguistically tailored lifestyle support, including gender specific groups, cooking classes, and community based activities, have proven effective in Sweden.
• Integrating health literacy into language and integration programs and developing culturally responsive, community-driven health policies are essential for addressing obesity inequities among immigrants.

## 1 Introduction

Overweight and obesity are among the most prevalent and preventable non-communicable diseases worldwide and have become major public health concerns in the Nordic region, affecting both native and immigrant populations (Østby and Gulbrandsen, 2022; Qureshi et al., 2020). According to World Health Organization (WHO) estimates, adult obesity rates in 2016 were high across the Nordic countries—Sweden 20.6 %, Denmark 19.7 %, Finland 22.2 %, Norway 23.1 %, and Iceland 21.9 % (WHO. WHO. World Health Organization 2017). These levels exceed the global average of 13.1 % but remain slightly below the European regional estimate of 23.3 % (WHO. WHO. World Health Organization 2017). The growing immigrant population in the region has further influenced national demographic and health profiles, including obesity prevalence patterns, as highlighted by the International Organization for Migration (IOM) and national demographic studies (Østby and Gulbrandsen, 2022; IOM 2022). Although immigration itself does not directly increase national obesity rates, immigrants’ health profiles often differ from those of native populations due to intersecting socioeconomic, cultural, behavioral, and environmental factors influencing diet, physical activity, and overall health behaviors (Østby and Gulbrandsen, 2022; IOM 2022; Ahmed et al., 2018).

Immigration to the Nordic countries has increased significantly over the past few decades, driven by a combination of economic migration, asylum seeking, and family reunification. This diverse immigrant population brings with it a range of socio-cultural practices and dietary habits that can influence health outcomes, including overweight and obesity. Evidence from Norway, Sweden, and Finland shows that the risk of overweight and obesity varies widely among immigrant subgroups, particularly among women of Middle Eastern and African origin, reflecting the combined effects of gender roles, socio-economic position, cultural norms, and dietary transitions after migration (Ahmed et al., 2018; Kjøllesdal et al., 2023 May). Several Nordic public health strategies, such as the Swedish Public Health Agency’s nutrition and physical-activity programs and Norway’s Action Plan for a Healthier Diet, have sought to address overweight and obesity through lifestyle promotion and culturally adapted information campaigns. However, health disparities persist, particularly among non-Western immigrants, where excess body weight is closely linked with higher rates of type 2 diabetes (T2D) and cardiovascular disease (Østby and Gulbrandsen, 2022; WHO. WHO. World Health Organization 2017).

Despite the significant public health impact of overweight and obesity, research focusing specifically on migration-related determinants of these conditions in the Nordic region remains limited compared with other health priorities (Kumar et al., 2022). While several individual studies have examined these issues among immigrants in Nordic countries, no comprehensive synthesis has yet mapped prevalence patterns, underlying determinants, and intervention efforts across this population. Available Nordic surveys indicate obesity rates of approximately 20 % in men and 27 % in women among immigrant groups, with 44 % of men and 34 % of women classified as overweight, based primarily on data from Norway and Sweden (Amiri, 2021). Globally, rates of overweight and obesity among adults have more than doubled since 1990. The World Health Organization (WHO) defines overweight as a body mass index (BMI) of 25 or higher and obesity as a BMI of 30 or higher. By 2022, 890 million adults—about 16 % of the global population—were classified as obese, marking obesity as a critical epidemic. A higher-than-optimal BMI contributed to an estimated 5 million deaths from non-communicable diseases (NCDs) in 2019 alone (WHO 2024). Overweight and obesity are major risk factors for several chronic conditions, including cardiovascular diseases, T2D, and certain cancers, highlighting the urgent need for effective, population-level prevention strategies (WHO 2024).

The causes of overweight and obesity are multifactorial. They stem from an imbalance between energy intake and expenditure but are shaped by a range of modifiable and contextual determinants (WHO 2024; Toselli et al., 2014; Skogberg et al., 2019; Mulugeta, 2020; Ismail et al., 2022). These determinants operate at several levels. Individual factors include genetic predisposition, psychological stressors such as depression or emotional eating, and behavioral habits related to diet and physical activity. Environmental and social influences include access to healthy foods, opportunities for physical activity, socioeconomic conditions that affect food choices, urban design, and policies regulating food pricing and advertising. Understanding how these layers interact is crucial for developing interventions that effectively prevent and manage obesity, particularly within diverse immigrant communities (Persson et al., 2014; Kjøllesdal et al., 2023; Menigoz et al., 2016). However, current evidence across the Nordic region remains fragmented, with few studies exploring how these determinants and intervention efforts intersect among immigrant populations. Because both structural and acculturation-related factors shape health trajectories after migration, a scoping review was conducted to systematically map the available evidence and identify key knowledge gaps concerning overweight and obesity among immigrant adults in the Nordic countries.

### 1.1 Aim and objectives of the study

The aim of this study is to map and synthesize existing research on overweight and obesity among adult immigrants in the Nordic countries. Specifically, the review seeks to identify evidence on the prevalence and distribution of overweight and obesity, examine the determinants and risk factors influencing these conditions, and summarize the interventions or prevention strategies that have been implemented, evaluated, or proposed within this population. The research questions guiding this review are:

• What evidence exists on the prevalence and distribution of overweight and obesity among adult immigrants in the Nordic countries?
• What determinants and risk factors have been reported to influence overweight and obesity in these populations?
• What interventions or prevention strategies have been implemented, evaluated, or proposed to address overweight and obesity among immigrant adults in the Nordic region?

## 2 Materials and methods

### 2.1 Study design

This study employed a scoping review methodology guided by the framework developed by Arksey and O’Malley (2005) and refined by Levac et al. (2010), further informed by the JBI Guidance for Conducting Systematic Scoping Reviews (Peters et al., 2015), and following the PRISMA Extension for Scoping Reviews (PRISMA-ScR) checklist to ensure transparency and methodological rigor. This design was chosen to comprehensively map existing evidence on overweight and obesity among immigrant adults in Nordic countries and to identify key themes, determinants, and interventions reported in the literature.

The review process included five stages:

a) Identifying the research question: mapping evidence on prevalence, determinants, and interventions addressing overweight and obesity among immigrant adults in Nordic countries.
b) Identifying relevant studies: comprehensive database searches were conducted to capture peer-reviewed studies published between 1 January 2013 and 31 December 2023.
c) Study selection: inclusion and exclusion criteria were applied, and two reviewers independently screened all records for relevance and completeness.
d) Charting the data: extracted information included study design, population characteristics, and findings related to prevalence, determinants, and interventions.
e) Collating and summarizing results: extracted data were synthesized descriptively to highlight overarching patterns, key determinants, and evidence gaps.

No formal quality appraisal was performed, consistent with scoping review methodology.

### 2.2 Study context

The review focused on studies reporting overweight and obesity measured by body mass index (BMI). Eligible studies included those presenting combined or average BMI values for adult immigrant populations (≥18 years) residing in the Nordic region. Only peer-reviewed research articles published in English were included, given the predominance of English in scientific databases.

### 2.3 Search strategy

A comprehensive search was performed across four databases—CINAHL, PsycINFO, PubMed, and Web of Science—using a strategy structured according to the PICOTS framework (Population, Intervention/Exposure, Comparator, Outcomes, Timing, and Setting) (Butler et al., 2017).

Relevant Medical Subject Headings (MeSH) and controlled vocabulary were reviewed in PubMed, but free-text keywords were ultimately applied to ensure cross-database consistency. A pilot search using alternative terms such as “foreign-born” and “ethnic minority” yielded no additional results, confirming the adequacy of the final search string: (Obesity OR overweight OR obese OR “high body mass index” OR “high BMI”)

AND (Immigrants OR immigration OR immigrant OR migrant OR migrants OR migration)

AND (Nordic countries OR Finland OR Finnish OR Sweden OR Swedish OR Denmark OR Danish OR Norway OR Norwegian OR Iceland OR Icelandic).

### 2.4 Data selection

Eligibility criteria are summarized in Table 1. Included studies were original, peer-reviewed research articles examining prevalence, determinants, risk factors, or interventions related to overweight and obesity among adult immigrants in Nordic countries. Studies focusing on children, descendants, or patients undergoing bariatric surgery were excluded.

**Table 1:** Inclusion and exclusion criteria.

Inclusion criteria	Exclusion criteria
Peer-reviewed research articles	Books, theses, editorials, reports, or reviews
Published 2013–2023	Published before 2013 or after 2023
Written in English	Written in other languages
Immigrant adults (≥ 18 years)	Children, adolescents, or descendants
Focused on overweight/obesity prevalence, determinants, or interventions	Focused on obesity therapy or bariatric surgery
Conducted in Nordic countries (Sweden, Norway, Finland, Denmark, Iceland)	Studies conducted outside the Nordic region
Non-pregnant adult populations	Pregnant women

### 2.5 Search outcomes

**Fig. 1:**
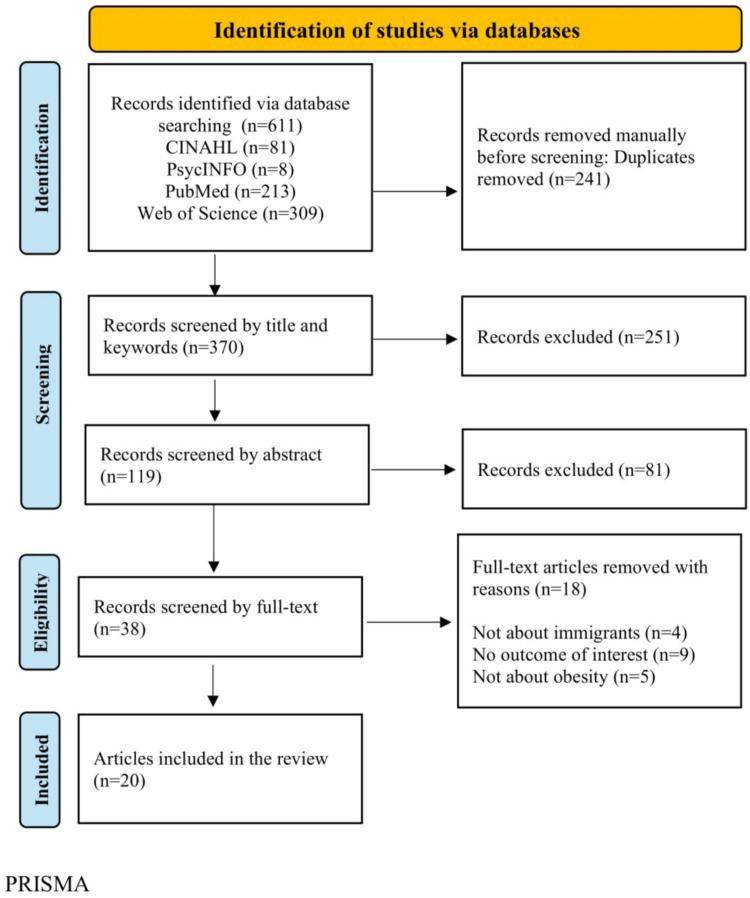
PRISMA.

A total of 611 records were identified from four electronic databases: CINAHL, PsycINFO, PubMed, and Web of Science. After 241 duplicate records were manually removed, 370 records remained and were screened based on titles and abstracts, resulting in the exclusion of 251 records. The remaining 119 records were imported into Zotero (Mac Version 6.0.37) for further management and screening. Following the abstract screening, 81 articles were excluded. Subsequently, 38 full-text articles were assessed for eligibility; 18 were excluded for not meeting the inclusion criteria. Ultimately, 20 studies met all eligibility requirements and were included in the final scoping review. The study selection process is illustrated in the PRISMA flow diagram (Fig. 1).

### 2.6 Data extraction and synthesis

Data extraction and synthesis followed the analytical guidance outlined by Pollock et al. (2023) for scoping reviews. Key study characteristics, including author, year, country, study design, population, and main findings on prevalence, determinants, and interventions, were recorded using a predefined data-charting form developed collaboratively by the review team. The extracted data were summarized descriptively and organized thematically in relation to the research questions. The synthesis focused on mapping the range, nature, and extent of evidence rather than evaluating study quality, consistent with the purpose of a scoping review.

## 3 Results

### 3.1 Study characteristics

Of the included studies, 16 utilized quantitative research methods (Qureshi et al., 2020; Ahmed et al., 2018; Kjøllesdal et al., 2023 May; Kjøllesdal et al., 2023; Bennet et al., 2016; Skogberg et al., 2016; Gele et al., 2016; Carlsson et al., 2014; Tennakoon et al., 2013; Siddiqui et al., 2017; Qureshi et al., 2022; Iversen et al., 2013; Al-Majdoub et al., 2020; Kinnunen et al., 2018; Gele and Mbalilaki, 2013; Bennet et al., 2022), while only four employed qualitative research methods (Persson et al., 2014; Sandstrom et al., 2015; Rieger et al., 2021; Olaya-Contreras et al., 2019). Ten studies were published in Norway, eight in Sweden, two in Finland, and none in Denmark or Iceland. There was a noted decrease in publications from 2019 to 2021, possibly attributed to the COVID-19 pandemic and shifting research priorities. However, the number of published studies has increased since 2022.

Among the qualitative studies, three out of four included only women, while the fourth included both men and women (Persson et al., 2014; Sandstrom et al., 2015; Rieger et al., 2021; Olaya-Contreras et al., 2019). Geographically, three of these qualitative studies were conducted in Sweden and one in Norway. Of the 16 quantitative studies, nine were conducted in Norway, five in Sweden, and two in Finland. Table 2 provides a detailed overview of the study characteristics.

**Table 2:** Characteristics of included studies.

Reference No, publication year, and country	Methodology	Population	Aim	Results
(Qureshi et al., 2020) Norway 2020	Quantitative: Secondary data analysis of a cross-sectional survey	4194 immigrants from 12 different countries	To examine associations between overweight/obesity and socio-demographic factors among immigrant groups in Norway.	53 % of participants were overweight/obese. Men were significantly more likely than women (60 % vs 44 %). Highest odds were among Turkish men (OR 5.9) and Somali women (OR 13.1). Age, country of origin, and marital status were strong predictors of overweight/obesity.
(Sandstrom et al., 2015) Sweden 2015	Qualitative: Semi-structured interview	Seven Bosnian women living in Malmö who migrated from rural or urban Bosnia during 1992–1993	To explore structural and individual factors acting as barriers or motivators for physical activity and a healthy lifestyle after migration	All seven women reported post-migration weight gain and acknowledged higher body weight compared with their pre-migration levels, consistent with prior evidence that migrant women in Sweden have slightly higher BMI than Swedish-born women. Reduced physical activity was attributed to colder climate, urban living, limited finances, family responsibilities, and lack of prior exercise experience. Despite these barriers, participants viewed physical activity as beneficial for mental health and weight control and expressed motivation to increase activity.
(Skogberg et al., 2016) Finland 201,601-Nov-25 8:17:00 PM	Quantitative: Cross-sectional study	921 migrants (344 Russians, 229 Somalis, 348 Kurds) and 892 Finns	To compare cardiovascular risk factors, including obesity, among Russian, Somali, and Kurdish migrants and the general Finnish population	Somali and Kurdish women had significantly higher obesity prevalence than Finnish women (PR 1.68, 95 % CI 1.40–2.03; PR 1.41, 95 % CI 1.15–1.72), whereas Somali men had lower obesity prevalence (PR 0.35, 95 % CI 0.17–0.71). Kurdish and Somali migrants also showed higher prevalence of hyperglycaemia and dyslipidaemia compared with the Finnish population, while Russian migrants’ profiles were largely similar to Finns.
(Carlsson et al., 2014) Sweden 2014	Quantitative: Cohort, Cross-sectional study	Main cohort: 4232 participants (777 immigrants and 3455 Swedes); Replication cohort: 26,777 participants (2016 immigrants and 24,761 Swedes) from the Malmö Diet and Cancer Study	To compare body mass index (BMI), waist circumference (WC), waist–hip ratio (WHR), sagittal abdominal diameter (SAD), waist–hip–height ratio (WHHR), and body fat percentage between immigrants and Swedish-born individuals	Finnish-born (BMI = 27.8 kg/m²; WC = 89.1 cm; WHR = 0.85; WHHR = 0.53), Middle Eastern-born (BMI = 32.7 kg/m²; WC = 97.9 cm; WHR = 0.88; WHHR = 0.58), and women from other non-European regions (BMI = 29.2 kg/m²; WC = 91.5 cm; WHR = 0.85; WHHR = 0.55) showed significantly higher anthropometric values compared with Swedish-born women (BMI = 26.4 kg/m²; WHHR = 0.50) (*p* < 0.001). Differences among men were smaller but significant for WHHR (Swedish-born = 0.54; Middle Eastern-born = 0.58), which most effectively detected obesity-related disparities between immigrant and Swedish-born groups.
(Siddiqui et al., 2017) Sweden 2017	Quantitative: Randomized Controlled Trial (RCT)	96 Iraqi-born adults in Malmö, Sweden, at high risk for Type 2 diabetes (50 intervention, 46 control)	To test the effect of a culturally adapted lifestyle intervention program on body weight, insulin sensitivity, and lipid profiles compared with usual care	After 3.9 months, the intervention group showed a significant reduction in body weight (–0.4 % per month, *p* = 0.004), BMI (–0.4 % per month, *p* = 0.004), and LDL cholesterol (–2.1 % per month, *p* = 0.036). Insulin sensitivity increased by 10.9 % per month (*p* = 0.005). 14.3 % of participants achieved ≥ 5 % weight loss vs none in the control group. No significant change was observed in caloric intake or HDL/triglycerides.
(Qureshi et al., 2022) Norway 2022	Quantitative: Cross-sectional study	5343 participants aged 45–79 years, including 1516 immigrants from 12 countries (Poland, Bosnia and Herzegovina, Kosovo, Turkey, Iraq, Iran, Afghanistan, Pakistan, Sri Lanka, Vietnam, Eritrea, and Somalia)	To investigate and compare functional ability, self-rated health, chronic diseases, and health behaviours—including overweight—among immigrants and the native Norwegian population	Adjusted for age, gender, and education, immigrants had significantly higher odds of overweight, chronic disease, and limitations in Activities of Daily Living (ADL) and Instrumental Activities of Daily Living (IADL) compared to Norwegians. Overweight prevalence was 65.4 % among immigrants and 57.6 % among Norwegians. Health disparities persisted despite adjustment for socioeconomic status.
(Kjøllesdal et al., 2023 May) Norway 2023	Quantitative: Cross-sectional study	3993 immigrants (2194 men, 1799 women) from 12 countries (Poland, Bosnia and Herzegovina, Kosovo, Turkey, Iraq, Iran, Afghanistan, Pakistan, Sri Lanka, Vietnam, Eritrea, and Somalia) with ≥2 years of residence in Norway	To assess health outcomes among immigrants in Norway by combinations of duration of residence, educational level, and Norwegian language proficiency	Immigrants had higher odds of obesity, diabetes, cardiovascular disease, and poor self-reported health than the general population. Obesity prevalence was higher among immigrants with long duration of residence (>10 years) and poor Norwegian language proficiency. Those with multiple disadvantages (long residence, low education, and poor Norwegian proficiency) had the highest odds of poor self-rated health (OR 10.13, 95 % CI 6.33–16.19) and cardiovascular disease (OR 8.72, 95 % CI 4.82–15.77) compared with Norwegian-born individuals.
(Iversen et al., 2013) Norway 2013	Quantitative: Cross-sectional study	3982 immigrants from five countries (Iran, Pakistan, Sri Lanka, Turkey, Vietnam) and 13,684 native Norwegians aged 30–60 years living in Oslo	To examine how acculturation, measured by Norwegian language proficiency, influences changes in BMI among immigrant groups relative to native Norwegians	Improved Norwegian language proficiency was associated with BMI changes converging toward native levels. Each step of better language proficiency reduced BMI increase by 0.71 kg/m² among Pakistani women. No significant effect was found for duration of residence. Turkish and Pakistani women showed the highest obesity levels; Vietnamese participants the lowest.
(Kinnunen et al., 2018) Finland 2018	Quantitative: Cross-sectional study	508 immigrant women aged 18–45 years (165 Russian, 164 Somali, 179 Kurdish) and 388 Finnish reference women from six Finnish municipalities (Helsinki, Espoo, Vantaa, Turku, Tampere, Vaasa)	To compare the prevalence of overweight (BMI ≥ 25 kg/m²) and abdominal obesity (waist-to-height ratio ≥ 0.5) between non-pregnant women of Russian, Somali, and Kurdish origin and Finnish women, and to examine the association between parity and overweight or abdominal obesity	Almost two-thirds of Kurdish (60.6 %) and Somali (63.8 %) women were overweight or obese versus 29.7 % of Finnish and 26.5 % of Russian women. After adjustment for sociodemographic and reproductive factors, Somali women had ≈ 3-fold and Kurdish women ≈ 2.6-fold odds of overweight compared with Finnish women, while Russian women had lower odds (OR 0.54, 95 % CI 0.33–0.89). Abdominal obesity was significantly more prevalent among Kurdish women (57.4 %) but not among Somali women after adjustment. Parity was significantly associated with higher odds of overweight (OR 2.6) and abdominal obesity (OR 3.6) only among Kurdish women.
(Gele and Mbalilaki, 2013) Norway 2013	Quantitative: Cross-sectional study	208 Somali immigrants aged 25 and over (115 women, 93 men)	To assess the prevalence of overweight and obesity and identify associated factors among Somali immigrants in Oslo	Overall, 50 % of participants were overweight or obese (35 % overweight, 15 % obese). Women had a significantly higher prevalence (66 %) than men (28 %) and higher mean BMI (27.4 vs. 23.6). Both generalized and abdominal obesity were strongly associated with longer duration of residence in Norway and low physical activity. Women were six times more likely than men to be overweight or obese (aOR 6.13, 95 % CI 2.81–13.4). Respondents with ≥14 years of residence were seven times more likely to be overweight/obese than those living ≤4 years (aOR 7.16, 95 % CI 2.14–23.8).
(Ahmed et al., 2018) Norway 2018	Quantitative: Comparable, cross-sectional studies	1330 Somali adults aged 20–69 years (1110 in Hargeisa, Somaliland; 220 in Oslo, Norway)	To compare the prevalence and predictors of overweight and obesity among Somalis in Oslo and Hargeisa	Obesity prevalence was substantially higher among Somali women in Oslo (44 %) than in Hargeisa (31 %), while men showed lower rates (9 % and 6 %, respectively). Women in Oslo had higher BMI, but similar waist circumference compared with women in Hargeisa. Higher BMI was associated with living in Oslo, older age, and being married among women.
(Kjøllesdal et al., 2023) Norway 2023	Quantitative: Cross-sectional study	4077 immigrants aged 16–66 years from 12 countries (Poland, Bosnia and Herzegovina, Kosovo, Turkey, Iraq, Iran, Afghanistan, Pakistan, Sri Lanka, Vietnam, Eritrea, and Somalia)	To examine the association between self-reported Norwegian language proficiency and multiple health outcomes, including overweight defined by BMI ≥ 25 kg/m².	The prevalence of overweight was 50.5 % among participants with good Norwegian proficiency, 55.7 % among those with medium proficiency, and 64.0 % among those with poor proficiency. Poor or medium language proficiency was associated with higher odds of overweight (OR = 1.20; 95 % CI: 1.03–1.40), even after adjusting for age, sex, and duration of residence.
(Persson et al., 2014) Sweden 2014	Qualitative: Focused ethnographic study	Twenty-six Somali women aged 17–67 years living in southern Sweden	To explore Somali women’s perceptions of health and physical activity following migration to Sweden	Participants acknowledged the importance of physical activity but lacked opportunities and knowledge to engage in it. Barriers included cultural and religious norms, cold climate, safety concerns, and loss of daily activity routines. These barriers contribute to sedentary behavior, which is known to elevate overweight and obesity risk.
(Bennet et al., 2016) Sweden 2016	Quantitative: Population-based cross-sectional study	1176 Iraqi-born and 688 Swedish-born adults aged 30–75 years	To identify BMI and waist-circumference cut-offs corresponding to equivalent levels of insulin sensitivity in Middle Eastern immigrants compared with native Swedes.	Insulin resistance occurred at lower BMI and waist-circumference thresholds among Iraqi participants than among Swedes. In normal-weight participants (BMI < 25 kg/m²), 21.2 % of Iraqis versus 9.3 % of Swedes were insulin-resistant. Equivalent insulin sensitivity in obese Swedes (BMI = 30 kg/m²) corresponded to BMI = 28.5 kg/m² in Iraqi men and 27.5 kg/m² in Iraqi women. Waist-circumference cut-offs equivalent to abdominal obesity in Swedes (≥ 94 cm for men, ≥ 80 cm for women) were 84 cm and 71 cm in Iraqi men and women, respectively.
(Gele et al., 2016) Norway 2016	Quantitative: Cross-sectional study	302 Somali women aged ≥25 years living in the Oslo area	To examine the association between duration of residence in Norway and type 2 diabetes (T2D) risk among Somali immigrant women	Among 302 Somali women aged ≥ 25 years, the mean BMI was 28.8 ± 5 kg/m². Overall, 43.2 % of participants were classified as overweight (BMI 25–29.9 kg/m²) and 35.2 % as obese (BMI ≥ 30 kg/m²). The mean waist circumference was 102 ± 13 cm, and 84 % had abdominal obesity (waist > 88 cm). Only 13.6 % reported engaging in at least 30 min of physical activity per day. Participants with higher BMI and waist circumference showed greater prevalence of metabolic risk factors, including hypertension and hyperglycaemia.
(Tennakoon et al., 2013) Norway 2013	Quantitative: Cross-sectional study	1145 Sri Lankans in Oslo (685 men, 460 women); 233 Tamils (103 men, 130 women) and 445 Sinhalese (143 men, 302 women) from Kandy, Sri Lanka	To compare lifestyle-related cardiovascular disease risk factors between Sri Lankans in Oslo and urban Tamils and Sinhalese in Kandy	Mean BMI (kg/m²): Oslo Sri Lankans men 25.3, women 26.1; Kandy Tamils men 22.4, women 23.8; Kandy Sinhalese men 22.0, women 23.1. Overweight/obesity ( %): Oslo women 38.3, men 31.9; Kandy Tamils women 33.9, men 14.6; Kandy Sinhalese women 25.9, men 12.3. Leisure-time physical inactivity ( %): Oslo men 51, women 55; Kandy Tamils men 65, women 85; Kandy Sinhalese men 66, women 87. Daily use of coconut/palm oil for cooking ( %): Kandy 98, Oslo 30. Daily fruit intake ( %): Kandy 46, Oslo 28.
(Al-Majdoub et al., 2020) Sweden 2020	Quantitative: cross-sectional study	93 Iraqi immigrants and 77 native Swedes aged 45–64 years living in Malmö	To examine metabolic and lifestyle differences between Iraqi immigrants and native Swedes to explain elevated T2D risk	Median BMI (kg/m²): Iraqi 29.3 [IQR 26.3–31.9]; Swedes 26.2 [IQR 23.8–29.7]. Obesity (BMI ≥ 30 kg/m²): 44.1 % vs 24.7 %. Hypertension: 32.3 % vs 58.4 %. Triglycerides (mmol/L): 1.4 vs 1.0; HDL (mmol/L): 1.01 vs 1.18. Physical inactivity: 63 % vs 60 %. Soft-drink consumption > once/week: 55 % vs 40 %. University education: 44.1 % vs 76.6 %. Twenty-six metabolites differed significantly between groups (*q* < 0.05), including higher levels of polyunsaturated acylcarnitines (14:2, 18:2) and fatty acid 18:2 in Iraqis and lower levels of saturated fatty acids (12:0, 14:0, 16:0, 18:1).
(Olaya-Contreras et al., 2019) Sweden 2019	Qualitative: Gender-specific focus group interviews	33 first-generation Iraqi immigrants in Malmö (19 women, 14 men) at high risk for type 2 diabetes (BMI > 28 kg/m² or prediabetes)	To explore perceptions, experiences, and barriers concerning lifestyle modification among Iraqi immigrants at risk of type 2 diabetes	Mean BMI (kg/m²): women 30.8, men 31.4. Participants showed awareness of healthy lifestyles but described multiple sociocultural barriers to weight control, including family expectations, portion-size norms, and irregular meal patterns. Low motivation, cold climate, and limited gender-appropriate exercise facilities further restricted physical activity, especially among women. Family involvement and support emerged as important facilitators for maintaining lifestyle change. No significant BMI difference between participants and non-participants (*p* > 0.05).
(Bennet et al., 2022) Sweden 2022	Quantitative: Secondary analysis of a RCT	92 Iraqi immigrants in Malmö, Sweden (48 intervention; 44 control), all overweight (BMI > 28 kg/m²) and at high risk of type 2 diabetes	To examine changes in plasma proneurotensin (Pro-NT) and determine whether baseline Pro-NT predicted weight loss during a culturally adapted lifestyle program	Baseline mean BMI (kg/m²): intervention group 31.0 ± 4.4, control 29.6 ± 3.6. Mean waist circumference (cm): men 108.2, women 100.7. After four months, body weight decreased by 2.5 kg in the intervention group versus an increase of 0.8 kg in controls (*p* = 0.028). BMI change correlated with baseline Pro-NT (β = 0.02; *p* = 0.041). Pro-NT levels rose by 28.2 pmol/L in the intervention group and 3.5 pmol/L in controls. Physical activity increased, and total fat intake declined slightly.
(Rieger et al., 2021) Norway 2021	Qualitative: Semi-structured interviews, and a focus group	15 Turkish immigrant women in Oslo aged 30–69 years	To explore beliefs and experiences related to body weight, nutrition, and exercise practices among Turkish immigrant women	Participants perceived Turkish women as more often overweight or obese than ethnic Norwegian women (previous data: 48 % vs. 11 %). They described cultural expectations around hospitality, shared meals, and social eating as major barriers to weight control. The Nordic climate and winter inactivity were linked to seasonal weight gain and low mood. Women expressed tension between traditional food practices and the “thin ideal,” reporting difficulty fitting Norwegian health and fitness norms. No BMI or anthropometric measurements were taken.

While prevalence data were dominant, a subset of studies focused on related determinants. Although all 20 studies addressed weight-related health, six studies (Bennet et al., 2016; Gele et al., 2016; Al-Majdoub et al., 2020; Bennet et al., 2022; Sandstrom et al., 2015; Rieger et al., 2021; Olaya-Contreras et al., 2019) did not directly quantify overweight or obesity prevalence but instead examined related metabolic, behavioral, or psychosocial determinants. Studies examining contextual or indirect mechanisms (Bennet et al., 2016; Gele et al., 2016; Al-Majdoub et al., 2020; Bennet et al., 2022; Sandstrom et al., 2015; Rieger et al., 2021; Olaya-Contreras et al., 2019) primarily investigated metabolic factors such as insulin sensitivity and diabetes risk (Bennet et al., 2016; Gele et al., 2016; Al-Majdoub et al., 2020; Bennet et al., 2022), as well as behavioral and psychosocial barriers related to lifestyle modification, physical activity, and cultural adaptation (Sandstrom et al., 2015; Rieger et al., 2021; Olaya-Contreras et al., 2019). These studies were therefore included as contextual evidence, as their findings help explain mechanisms that contribute indirectly to overweight and obesity among immigrant adults in the Nordic region (Bennet et al., 2016; Gele et al., 2016; Al-Majdoub et al., 2020; Bennet et al., 2022; Sandstrom et al., 2015; Rieger et al., 2021; Olaya-Contreras et al., 2019). Overall, the included studies largely represent the major non-Western immigrant populations residing in the Nordic region—particularly Somali (Ahmed et al., 2018; Persson et al., 2014; Gele et al., 2016; Gele and Mbalilaki, 2013), Iraqi (Bennet et al., 2016; Siddiqui et al., 2017; Al-Majdoub et al., 2020; Bennet et al., 2022; Olaya-Contreras et al., 2019), Kurdish (Skogberg et al., 2016; Kinnunen et al., 2018), and Turkish groups (Qureshi et al., 2020; Iversen et al., 2013; Rieger et al., 2021)—which correspond to the largest immigrant communities in Norway and Sweden.

### 3.2 Prevalence of overweight and obesity

Table 3 summarizes the prevalence of overweight and obesity among immigrant adults in Nordic countries. Quantitative studies explicitly reporting prevalence or BMI comparisons included (Qureshi et al., 2020; Ahmed et al., 2018; Kjøllesdal et al., 2023 May; Kjøllesdal et al., 2023; Skogberg et al., 2016; Carlsson et al., 2014; Qureshi et al., 2022; Iversen et al., 2013; Al-Majdoub et al., 2020; Kinnunen et al., 2018; Gele and Mbalilaki, 2013). Other studies reported BMI or body-weight information without presenting population-level prevalence estimates (Bennet et al., 2016; Gele et al., 2016; Siddiqui et al., 2017; Bennet et al., 2022; Sandstrom et al., 2015; Rieger et al., 2021; Olaya-Contreras et al., 2019). Across the prevalence studies, immigrants from the Middle East and Somali women consistently exhibited higher obesity rates than the native populations.

**Table 3:** Prevalence of overweight and obesity among immigrant adults in Nordic countries.

Reference (No.)	Country / Year	Prevalence of Overweight and Obesity
(Qureshi et al., 2020)	Norway 2020	53 % of the sample was overweight or obese (men 60 %, women 44 %); highest prevalence observed among Turkish (67 %) and lowest among Vietnamese (27 %) participants.
(Sandstrom et al., 2015)	Sweden 2015	All seven Bosnian women reported post-migration weight gain, indicating an increased risk of overweight and obesity after relocation.
(Skogberg et al., 2016)	Finland 2016	Men: Finnish 18.5 %, Russian 17.1 %, Somali 5.0 %, Kurdish 20.7 %. Women: Finnish 20.6 %, Russian 21.5 %, Somali 54.1 %, Kurdish 33.2 %. Somali and Kurdish women had the highest obesity prevalence (PR 1.68 and 1.41 vs. Finnish women).
(Carlsson et al., 2014)	Sweden 2014	Average BMI by nationality and gender (Main Study / Malmö Diet and Cancer Study): Swedish men 26.8 / 26.2, women 26.4 / 25.3; Finnish men 27.5 / 27.3, women 27.8 / 26.0; Eastern European men 28.0 / 26.7, women 26.9 / 26.5; Southern/Western Europe + North America men 27.3, women 27.7; Middle Eastern men 29.3 / 25.3, women 32.7 / 27.8; Rest of World men 27.5 / 26.4, women 29.2 / 26.4; Danish men 27.2, women 25.2. Overall, Finnish-, Middle Eastern-, and non-European women showed the highest obesity indicators.
(Siddiqui et al., 2017)	Sweden 2017	Mean BMI 31.0 (intervention) and 29.6 (control) at baseline. All participants classified as overweight or obese (BMI ≥ 28 kg/m²) and at high risk for Type 2 diabetes.
(Qureshi et al., 2022)	Norway 2022	65.4 % of immigrants were overweight or obese compared to 57.6 % of the Norwegian reference population. Overweight prevalence varied widely among immigrant groups (29.2–87.7 %).
(Kjøllesdal et al., 2023 May)	Norway 2023	The prevalence of obesity among immigrants was 14.4 %, compared with 13.1 % in the general Norwegian population. Obesity rates were particularly high among immigrants with poor Norwegian language proficiency (20.3 %) and long duration of residence (>10 years, 16.4 %), compared with those with fair/good proficiency (13.8 %) or shorter residence (11.3 %).
(Iversen et al., 2013)	Norway 2013	Obesity rates: Norwegian women 11 %, men 14 %; Turkish women 47 %, men 23 %; Sri Lankan women 19 %, men 6 %; Iranian women 11 %, men 13 %; Pakistani women 35 %, men 25 %; Vietnamese women 4 %, men 3 %.
(Kinnunen et al., 2018)	Finland 2018	Obesity rates: Finnish 9.8 %, Russian 8.0 %, Somali 30.9 %, Kurdish 19.5 %. Overall prevalence of overweight (BMI ≥ 25 kg/m²): Finnish 29.7 %, Russian 26.5 %, Somali 63.8 %, Kurdish 60.6 %. Abdominal obesity (waist-to-height ≥ 0.5): Finnish 26.7 %, Russian 23.3 %, Somali 44.3 %, Kurdish 57.4 %.
(Gele and Mbalilaki, 2013)	Norway 2013	Overweight/obesity: Women 66 %, Men 28 %. Abdominal obesity: Women 53 %, Men 28 %. Mean BMI: 27.4 (women) and 23.6 (men).
(Ahmed et al., 2018)	Norway 2018	Oslo: Females 44.1 %, Males 9.2 %. Hargeisa: Females 31.3 %, Males 6.2 %. Central obesity (waist circumference ≥ 88 cm for women, ≥ 102 cm for men) was 50.9 % among women in Oslo and 49.5 % among women in Hargeisa; 31.8 % of men in Oslo and 6.2 % of men in Hargeisa were centrally obese.
(Kjøllesdal et al., 2023)	Norway 2023	Overweight prevalence: Good proficiency 50.5 %, Medium 55.7 %, Poor 64.0 % (BMI ≥ 25 kg/m²).
(Persson et al., 2014)	Sweden 2014	This study qualitatively explores contextual, behavioral, and cultural factors that contribute to physical inactivity and increased obesity risk among Somali women living in Sweden (BMI not directly measured).
(Bennet et al., 2016)	Sweden 2016	Obesity prevalence: Iraqi women 37.1 %, Iraqi men 33.4 %; Swedish women 21.8 %, Swedish men 21.3 %. Insulin resistance was observed even among normal-weight Iraqis (21.2 %) and overweight Iraqis (45.2 %).
(Gele et al., 2016)	Norway 2016	Overweight: 43.2 % (25–29.9 kg/m²); Obesity: 35.2 % (≥30 kg/m²); Mean BMI: 28.8 kg/m² (SD 5). Abdominal obesity: 84 % (WC > 88 cm); Mean waist circumference: 102 cm (SD 13).
(Tennakoon et al., 2013)	Norway 2013	Overweight/obesity ( %): Oslo Sri Lankans – women 38.3, men 31.9; Kandy Tamils – women 33.9, men 14.6; Kandy Sinhalese – women 25.9, men 12.3. Mean BMI (kg/m²): Oslo 26.1 (women), 25.3 (men); Kandy Tamils 23.8 (women), 22.4 (men); Kandy Sinhalese 23.1 (women), 22.0 (men).
(Al-Majdoub et al., 2020)	Sweden 2020	Obesity (BMI ≥ 30 kg/m²): Iraqi 44.1 %, Swedes 24.7 %. Median BMI (kg/m²): Iraqi 29.3 [IQR 26.3–31.9]; Swedes 26.2 [IQR 23.8–29.7]. Hypertension: 32.3 % vs 58.4 %.
(Olaya-Contreras et al., 2019)	Sweden 2019	Mean BMI (kg/m²): women 30.8, men 31.4 — indicative of obesity. All participants were classified as overweight or obese (BMI > 28 kg/m²) based on inclusion criteria.
(Bennet et al., 2022)	Sweden 2022	Mean BMI (kg/m²): 31.0 (intervention) and 29.6 (control); all participants classified as overweight or obese (BMI > 28 kg/m²). Mean waist circumference: men 108.2 cm; women 100.7 cm.
(Rieger et al., 2021)	Norway 2021	Qualitative findings from Turkish immigrant women indicated a shared perception that Turkish women are more commonly overweight or obese than Norwegian women. Participants expressed concern about overweight and obesity in their community and described cultural and environmental barriers to weight control, such as hospitality norms and limited winter activity. No direct BMI measurement was conducted.

Among Somali immigrant women, obesity prevalence ranged from 30.9 % to 54.1 %, markedly higher than that of the general population (Ahmed et al., 2018; Skogberg et al., 2016; Kinnunen et al., 2018). The 30.9 % prevalence among younger Somali women aged 18 to 45 years highlighted group-specific differences within this population (Kinnunen et al., 2018). In comparison, obesity rates among general female populations ranged between 9.8 % and 20.6 % (Skogberg et al., 2016; Kinnunen et al., 2018).

Across Norwegian studies, the overall prevalence of overweight and obesity among immigrants ranged from 53 % to 65 %, exceeding that of native Norwegians. Several studies reported notable gender differences, with obesity rates approximately twice as high among women as among men (Iversen et al., 2013; Gele and Mbalilaki, 2013). High levels of abdominal obesity were also reported among Somali women, with more than half exceeding recommended waist-circumference thresholds (Ahmed et al., 2018; Gele et al., 2016).

In contrast, immigrants from Russia, other European regions, and high-income countries generally exhibited obesity prevalence rates similar to those of host-country populations (Qureshi et al., 2020; Kjøllesdal et al., 2023 May; Kjøllesdal et al., 2023; Skogberg et al., 2016; Carlsson et al., 2014; Qureshi et al., 2022; Kinnunen et al., 2018). However, Iraqi immigrants showed substantially higher obesity rates, with Iraqi women at 37.1 % and Iraqi men at 33.4 %, compared with 21.8 % and 21.3 % among the respective Swedish groups (Bennet et al., 2016; Al-Majdoub et al., 2020). Similarly, Kurdish immigrant women demonstrated obesity prevalence between 19.5 % and 33.2 %, whereas Finnish women showed rates between 9.8 % and 20.6 % (Skogberg et al., 2016; Kinnunen et al., 2018). Among Kurdish men, obesity prevalence was 20.7 % compared with 18.5 % among Finnish men (Skogberg et al., 2016).

No prevalence data were identified for Denmark or Iceland within the inclusion period, and most studies were limited to Norway, Sweden, and Finland. This imbalance highlights remaining geographic and demographic evidence gaps in the Nordic literature.

### 3.3 Determinants and risk factors of overweight and obesity

Determinants and risk factors identified across the included studies reflected a complex interaction between individual, structural, and sociocultural influences shaping weight outcomes among immigrant adults in the Nordic region. At the individual level, limited physical activity, high-energy dietary intake, and low health literacy were consistently reported as key behavioral drivers of overweight and obesity (Qureshi et al., 2020; Ahmed et al., 2018; Bennet et al., 2016; Gele et al., 2016; Carlsson et al., 2014; Siddiqui et al., 2017; Qureshi et al., 2022; Al-Majdoub et al., 2020; Kinnunen et al., 2018; Gele and Mbalilaki, 2013; Bennet et al., 2022). Several studies also identified metabolic and biological risk factors, including insulin resistance, hyperglycaemia, dyslipidaemia, hypertension, and abnormal triglyceride or HDL levels, reporting that post-migration dietary and lifestyle changes were linked to weight gain and cardiometabolic vulnerability (Bennet et al., 2016; Gele et al., 2016; Al-Majdoub et al., 2020). The clustering of these risk markers among Middle Eastern and African immigrant groups reflected the combined effects of nutritional transition, reduced physical activity, and constrained access to preventive care. Most evidence focused on Middle Eastern and African immigrants, while major migrant groups from Eastern Europe and Asia—such as Polish, Thai, and Filipino populations—were seldom represented.,

Beyond individual behaviors, the structural determinants of obesity among immigrants were strongly tied to socioeconomic inequality, low educational attainment, and limited employment opportunities (Qureshi et al., 2020; Skogberg et al., 2016; Tennakoon et al., 2013; Qureshi et al., 2022; Al-Majdoub et al., 2020; Kinnunen et al., 2018). Economic hardship, time constraints from work and family responsibilities, and reliance on inexpensive calorie-dense foods emerged as recurring themes shaping dietary behaviors and physical activity levels. Studies also emphasized the impact of language barriers, low acculturation, and inadequate access to culturally adapted health information, which together limited engagement with preventive health services and hindered understanding of dietary and exercise recommendations (Kjøllesdal et al., 2023 May; Kjøllesdal et al., 2023; Iversen et al., 2013). Analyses also linked neighborhood deprivation, housing conditions, and restricted healthcare access to higher rates of excess weight and chronic disease among immigrants (Qureshi et al., 2020; Kjøllesdal et al., 2023; Qureshi et al., 2022). Collectively, the studies reported that socioeconomic disadvantage and communication barriers operated as structural constraints reinforcing obesogenic environments in the Nordic context.

The duration of residence in the host country emerged as a consistent cross-cutting determinant. Longer residence was frequently associated with higher obesity prevalence, reflecting dietary westernization, reduced physical labor, and increased sedentary urban lifestyles (Ahmed et al., 2018; Kjøllesdal et al., 2023 May; Gele et al., 2016; Gele and Mbalilaki, 2013). This pattern was particularly visible among Somali and South Asian immigrants, whose earlier active lifestyles in their countries of origin contrasted sharply with reduced mobility and increased reliance on convenience foods following migration. Several studies also observed that the relationship between residence duration and obesity differed by gender, with women experiencing more pronounced weight increases after migration, possibly due to lower workforce participation and limited access to recreational physical activity.

Gender-specific and sociocultural influences were prominent across qualitative and mixed-methods studies (Persson et al., 2014; Siddiqui et al., 2017; Gele and Mbalilaki, 2013; Sandstrom et al., 2015; Rieger et al., 2021; Olaya-Contreras et al., 2019). Cultural expectations surrounding hospitality, shared meals, and food generosity fostered social norms of overeating, while traditional gender roles and religious norms often restricted women’s participation in mixed-gender or outdoor exercise. Participants in Sweden and Norway described difficulties balancing household responsibilities, child-rearing, and paid work, which collectively limited time for physical activity. Seasonal and climatic factors—particularly long, dark winters and cold weather—further constrained outdoor activity and were linked to emotional eating and weight gain during winter months (Persson et al., 2014; Sandstrom et al., 2015; Rieger et al., 2021).

Across the included studies, obesity among immigrant adults in the Nordic region was associated with overlapping behavioral, metabolic, sociocultural, and structural factors. Cultural adaptation, language proficiency, socioeconomic position, and duration of residence were the most consistently reported correlates of excess weight.

Table 4.

**Table 4:** Determinants and risk factors for overweight and obesity among immigrant adults in Nordic countries.

Reference (No.)	Country / Year	Determinants and Risk Factors
(Qureshi et al., 2020)	Norway / 2020	Gender, age, marital status, country of origin, and self-reported diabetes were significantly associated with higher odds of overweight/obesity. No association found for education, physical activity, or length of residence.
(Sandstrom et al., 2015)	Sweden / 2015	Barriers included colder climate, urban lifestyle, limited financial resources, family and work priorities, and lack of earlier experience with planned physical activity; motivational factors included perceived benefits for mental health and weight loss.
(Skogberg et al., 2016)	Finland / 2016	Variations in obesity and metabolic risk were linked to ethnic background, sex, and migration-related lifestyle factors. Somali and Kurdish migrants showed a higher prevalence of hyperglycaemia and dyslipidaemia but lower rates of hypertension compared with the Finnish population.
(Carlsson et al., 2014)	Sweden / 2014	Integration challenges and lifestyle adaptation differences influencing diet and physical activity; environmental and socio-cultural factors affecting anthropometric outcomes; possible effects of migration-related sedentary patterns and urbanization.
(Siddiqui et al., 2017)	Sweden / 2017	Cultural and socioeconomic barriers limiting physical activity and dietary change; gender norms restricting women’s exercise; limited health literacy; economic constraints for gym access and healthy foods.
(Qureshi et al., 2022)	Norway / 2022	Economic inequalities, lower educational attainment, limited access to healthcare, language barriers, cultural dietary patterns, risky occupations, and low physical activity. Chronic disease burden and functional limitations were also linked to overweight.
(Kjøllesdal et al., 2023 May)	Norway / 2023	Longer duration of residence, poor Norwegian language proficiency, and low education were strongly associated with higher obesity rates. Socioeconomic disadvantage, health literacy limitations, cultural dietary habits, social isolation, and restricted access to preventive health services contributed to elevated obesity risk.
(Iversen et al., 2013)	Norway / 2013	Limited Norwegian language proficiency, cultural dietary habits, socioeconomic disadvantage, and gender-specific barriers to physical activity contributed to higher obesity risk, particularly among Turkish and Pakistani women. Length of residence did not affect BMI changes, while language proficiency showed strong protective effects.
(Kinnunen et al., 2018)	Finland / 2018	Economic hardship, low education and employment levels, high parity, and sociocultural norms influencing diet and physical activity contributed to excess weight among Somali and Kurdish women. Language barriers, limited access to safe exercise environments, and post-migration lifestyle changes further reinforced risk.
(Gele and Mbalilaki, 2013)	Norway / 2013	Duration of residence exceeding 14 years, low levels of physical activity, high-calorie dietary patterns, and a sedentary post-migration lifestyle were major determinants of overweight and obesity. Cultural norms associating larger body size with health and success, together with limited access to gender-sensitive exercise facilities, further increased risk among Somali women.
(Ahmed et al., 2018)	Norway / 2018	Nutritional transition after migration, characterized by adoption of Western dietary patterns, reduced physical activity, and sedentary urban lifestyles. In Oslo, higher BMI was associated with older age, being married, and residence in a high-income environment; in Hargeisa, lower educational level predicted higher BMI.
(Kjøllesdal et al., 2023)	Norway / 2023	Poor or medium Norwegian language proficiency increased the likelihood of being overweight (OR = 1.20; 95 % CI: 1.03–1.40). Limited language proficiency reduced access to health information, constrained understanding of preventive advice, and lowered engagement in physical activity, indirectly increasing overweight risk.
(Persson et al., 2014)	Sweden / 2014	Cultural norms restricting women’s participation in mixed-gender physical activity, lack of female-only facilities, safety and climate barriers, and continuation of high-calorie traditional diets after migration. Loss of everyday mobility (e.g., walking and manual chores) increased sedentary time, heightening obesity risk.
(Bennet et al., 2016)	Sweden / 2016	Ethnic and gender differences in fat distribution and metabolism were identified. Waist circumference showed a stronger association with insulin resistance among Iraqi men compared to Swedes, indicating higher visceral adiposity. Elevated triglyceride levels, male gender, and larger waist circumference were associated with reduced insulin sensitivity in the Iraqi group.
(Gele et al., 2016)	Norway / 2016	Higher BMI and waist circumference were associated with increased metabolic risk factors such as hypertension and hyperglycaemia. Longer residence in Norway (> 10 years) was independently associated with a twofold higher diabetes risk (OR 2.16; 95 % CI 1.08–4.32), reflecting the influence of acculturation on body composition. Low physical activity was common (86.4 % reported < 30 min/day).
(Tennakoon et al., 2013)	Norway / 2013	Urbanization and acculturation influencing diet composition and physical activity; Kandy participants consumed predominantly saturated fats (> 90 % used coconut/palm oil daily), while Oslo participants used more unsaturated oils and soft margarines. Physical inactivity was higher among Kandy women (85–87 %).
(Al-Majdoub et al., 2020)	Sweden / 2020	Higher obesity rates among Iraqi immigrants associated with lower educational attainment, higher triglycerides (1.4 mmol/L vs 1.0), lower HDL (1.01 mmol/L vs 1.18), greater soft-drink consumption (55 % vs 40 %), and similar physical inactivity (63 % vs 60 %). Metabolite profiling showed elevated polyunsaturated fatty acids (18:2) and acylcarnitines (14:2, 18:2) but lower saturated fatty acids, suggesting dietary differences linked to migration and acculturation.
(Olaya-Contreras et al., 2019)	Sweden / 2019	Cultural and family norms influencing food preparation, large portion sizes, frequent intake of fried foods and sweet beverages, irregular meals, and gender-related constraints on physical activity. Low motivation and environmental factors (cold climate, household workload) contributed to sedentary lifestyle.
(Bennet et al., 2022)	Sweden / 2022	Sedentary lifestyle, high total and saturated fat intake (> 40 % of total calories), limited access to culturally appropriate exercise opportunities, and social norms reinforcing high-calorie food consumption.
(Rieger et al., 2021)	Norway / 2021	Cultural norms of hospitality and generosity, frequent social eating, limited outdoor activity due to cold climate and long winter darkness, emotional eating during winter months, and tension between traditional and Western body ideals.

### 3.4 Interventions to address overweight and obesity

Across the reviewed literature, only a few studies empirically tested interventions designed to address overweight and obesity among immigrant adults in the Nordic countries, while most publications proposed culturally and contextually adapted strategies without formal evaluation (Siddiqui et al., 2017; Bennet et al., 2022). The intervention studies were conducted exclusively in Sweden and primarily targeted Middle Eastern immigrant populations at elevated risk of type 2 diabetes (Bennet et al., 2016; Siddiqui et al., 2017; Al-Majdoub et al., 2020; Bennet et al., 2022), whereas research from Norway and Finland mainly presented policy-oriented recommendations and community-level strategies aimed at specific demographic groups, particularly women of reproductive age and long-term residents facing linguistic or socioeconomic barriers (Ahmed et al., 2018; Kjøllesdal et al., 2023 May; Persson et al., 2014; Kjøllesdal et al., 2023; Skogberg et al., 2016; Gele et al., 2016; Tennakoon et al., 2013; Qureshi et al., 2022; Iversen et al., 2013; Kinnunen et al., 2018; Gele and Mbalilaki, 2013; Sandstrom et al., 2015; Rieger et al., 2021; Olaya-Contreras et al., 2019). No empirical intervention studies were identified from Norway, Finland, Denmark, or Iceland, indicating that intervention evidence remains geographically concentrated in Sweden.

In Sweden, two culturally adapted lifestyle interventions among Iraqi immigrants reported significant short-term effects on weight reduction and metabolic outcomes (Siddiqui et al., 2017; Bennet et al., 2022). A randomized controlled trial (Siddiqui et al., 2017) involving a four-month culturally adapted program with gender-specific sessions, Arabic-speaking coaches, cooking classes, and empowerment workshops reported an average body-weight reduction of 5 % or more in 14 % of participants, alongside measurable improvements in insulin sensitivity and lipid profiles. A follow-up analysis from the same cohort (Bennet et al., 2022) found sustained benefits, with an average weight decrease of 2.5 kg among intervention participants compared with a 0.8 kg gain in the control group after four months. These results represent the most robust empirical data available in the Nordic region on structured, culturally responsive, and linguistically tailored lifestyle-modification programs for immigrant populations.

Complementary research reported additional physiological data supporting the need for culturally adapted interventions. A population-based study (Bennet et al., 2016) found that equivalent insulin sensitivity in obese Swedes corresponded to lower BMI and waist-circumference values among Iraqi men and women, indicating that standard European obesity cutoffs may underestimate metabolic risk in Middle Eastern groups. A later study (Al-Majdoub et al., 2020) expanded on this by documenting distinctive metabolite profiles—such as elevated triglycerides and polyunsaturated fatty acids—among Iraqi immigrants. Together, these findings suggested physiological variability among immigrant populations relevant to intervention tailoring.

Beyond the empirically tested Swedish interventions, most Nordic studies proposed policy and practice recommendations emphasizing the creation of supportive environments that enable healthy lifestyles. Norwegian studies (Ahmed et al., 2018; Kjøllesdal et al., 2023 May; Kjøllesdal et al., 2023; Gele et al., 2016; Tennakoon et al., 2013; Qureshi et al., 2022; Iversen et al., 2013; Gele and Mbalilaki, 2013; Rieger et al., 2021) consistently called for integrating culturally sensitive nutrition education and physical-activity promotion into immigrant health frameworks. Several highlighted the need to link language training with health-literacy initiatives to enhance communication and self-management capacities (Kjøllesdal et al., 2023 May; Kjøllesdal et al., 2023; Iversen et al., 2013). Others emphasized early post-migration counseling on diet and exercise (Gele and Mbalilaki, 2013) and community-based programs for women, designed to accommodate cultural norms around modesty and family-centered roles (Ahmed et al., 2018; Persson et al., 2014; Rieger et al., 2021). The importance of accessible recreational spaces and safe female-only exercise environments was repeatedly reported across both Norwegian and Swedish qualitative studies (Persson et al., 2014; Sandstrom et al., 2015; Rieger et al., 2021; Olaya-Contreras et al., 2019).

In Finland, recommendations focused on embedding culturally adapted health promotion within existing public-health infrastructures. Maternal and child-health clinics were identified as effective entry points for delivering nutritional and physical-activity guidance to migrant women of childbearing age (Skogberg et al., 2016; Kinnunen et al., 2018). Broader policy suggestions included integrating obesity surveillance and lifestyle counseling into national preventive frameworks and addressing socioeconomic determinants of inactivity and unhealthy diets (Skogberg et al., 2016; Kinnunen et al., 2018). Similarly, Norwegian and Swedish authors recommended that obesity prevention strategies incorporate community health workers, immigrant organizations, and local faith or women’s groups to ensure cultural relevance and sustained participation (Persson et al., 2014; Sandstrom et al., 2015; Rieger et al., 2021; Olaya-Contreras et al., 2019).

Although not empirically evaluated within the included studies, Sweden’s national Physical Activity on Prescription program (FaR) was repeatedly cited as a transferable model for embedding structured physical-activity recommendations into routine care (Al-Majdoub et al., 2020; Sandstrom et al., 2015). Its emphasis on individualized, evidence-based exercise prescriptions and subsidized access to training facilities aligned closely with the culturally tailored and gender-sensitive approaches advocated across Nordic research.

Across the included studies, culturally tailored, gender-responsive, and linguistically adapted interventions—especially when grounded in community contexts—were most frequently associated with improved weight management and lifestyle behaviors among immigrant populations in the Nordic region. Empirical results from Sweden (Siddiqui et al., 2017; Bennet et al., 2022) reported measurable short-term effects, while proposed frameworks from Norway and Finland emphasized preventive counseling, health education, and language training within broader integration and maternal-health programs (Kjøllesdal et al., 2023 May; Kjøllesdal et al., 2023; Skogberg et al., 2016; Gele et al., 2016; Tennakoon et al., 2013; Iversen et al., 2013; Kinnunen et al., 2018; Gele and Mbalilaki, 2013). Studies (Bennet et al., 2016; Al-Majdoub et al., 2020) also identified group-specific metabolic profiles, such as distinctive triglyceride and fatty-acid patterns, highlighting physiological variability among immigrant groups.

Table 5.

**Table 5:** Suggested interventions for overweight and obesity among immigrant adults in Nordic countries.

Reference (No.)	Country / Year	Suggested Interventions
(Qureshi et al., 2020)	Norway / 2020	The findings highlight the need for culturally adapted obesity prevention strategies targeting high risk groups such as Somali, Pakistani, Kosovan, and Iraqi women, and Turkish and Polish men. No intervention was implemented.
(Sandstrom et al., 2015)	Sweden / 2015	The study recommends improving access to affordable and culturally appropriate physical activity opportunities, promoting workplace exercise initiatives, and implementing Physical Activity on Prescription (FaR) in collaboration with immigrant organizations to support sustainable participation among migrant women. No intervention was implemented.
(Skogberg et al., 2016)	Finland / 2016	The findings highlight the need for culturally tailored cardiovascular and obesity prevention programs addressing group specific metabolic risk patterns. Targeted health promotion efforts for migrant populations, particularly Somali and Kurdish women, should be incorporated into Finland’s preventive health frameworks. No intervention was implemented.
(Carlsson et al., 2014)	Sweden / 2014	The study recommends integrating WHHR as a complementary obesity indicator in public health monitoring and preventive strategies to better identify at risk immigrant populations. Broader implications include inclusive community planning and culturally sensitive approaches to address social and lifestyle disparities. No intervention was implemented.
(Siddiqui et al., 2017)	Sweden / 2017	The intervention involved a four month culturally adapted lifestyle program including seven gender specific sessions, a cooking class, Arabic speaking coaches, empowerment activities, education on healthy eating and physical activity, and financial support for training gear. It produced significant reductions in body weight and LDL cholesterol and improved insulin sensitivity, and is recommended for integration into primary health care practice for high risk immigrant groups.
(Qureshi et al., 2022)	Norway / 2022	The findings emphasize strengthening data driven public health policies addressing socioeconomic and cultural barriers through community based programs for older immigrants focusing on health literacy, culturally appropriate nutrition, and physical activity promotion. Incorporating immigrant health surveillance including ADL, IADL, BMI, and chronic conditions into national monitoring systems is recommended. No intervention was implemented.
(Kjøllesdal et al., 2023 May)	Norway / 2023	The study recommends strengthening integration policies linking language education and health promotion through sustained culturally tailored programs. Expanded health literacy initiatives and preventive care outreach for long term immigrants with limited host language proficiency are needed. No intervention was implemented.
(Iversen et al., 2013)	Norway / 2013	The findings support combining acculturation and integration programs that include language training and culturally adapted health education. Gender sensitive community health initiatives promoting nutrition awareness and physical activity are encouraged. No intervention was implemented.
(Kinnunen et al., 2018)	Finland / 2018	The findings suggest utilizing existing public health infrastructures such as maternity and child health clinics to deliver culturally tailored nutrition and physical activity counseling. Community based programs addressing reproductive health and weight management for migrant women of childbearing age are recommended. No intervention was implemented.
(Gele and Mbalilaki, 2013)	Norway / 2013	The study recommends implementing culturally sensitive physical activity programs and early post migration health counseling on diet and exercise. Collaboration with community organizations and urban planners could enhance access to recreational spaces for immigrant women. No intervention was implemented.
(Ahmed et al., 2018)	Norway / 2018	The results highlight the need for long term culturally adapted prevention strategies promoting balanced nutrition and active lifestyles among Somali adults, especially women. Integrating health education, dietary counseling, and community based physical activity programs within immigrant and urban health frameworks is recommended. No intervention was implemented.
(Kjøllesdal et al., 2023)	Norway / 2023	The study emphasizes strengthening immigrant focused language and health education programs. Norwegian language training should be integrated with culturally adapted nutrition and physical activity initiatives to improve communication and lifestyle adoption. No intervention was implemented.
(Persson et al., 2014)	Sweden / 2014	The study recommends community based and culturally adapted programs promoting physical activity in safe female only environments. Involving Somali community leaders and health workers in intervention design can enhance cultural relevance. No intervention was implemented.
(Bennet et al., 2016)	Sweden / 2016	The findings indicate a need to integrate ethnicity specific BMI and waist circumference cutoffs into clinical screening for metabolic syndrome and diabetes prevention. Developing culturally sensitive prevention programs focusing on early detection and lifestyle modification in Middle Eastern immigrants is recommended. No intervention was implemented.
(Gele et al., 2016)	Norway / 2016	The study highlights the need for culturally adapted diabetes prevention programs promoting physical activity and healthy dietary habits among Somali immigrant women. No intervention was implemented.
(Tennakoon et al., 2013)	Norway / 2013	The findings emphasize culturally adapted nutrition education focusing on reducing saturated fat intake and increasing physical activity. No intervention was implemented.
(Al-Majdoub et al., 2020)	Sweden / 2020	The findings call for detailed studies on dietary patterns to inform long term nutritional guidance programs tailored to immigrant populations. Nutritional and metabolic monitoring is needed to understand diet related risks among Middle Eastern immigrants. No intervention was implemented.
(Olaya-Contreras et al., 2019)	Sweden / 2019	No additional intervention was implemented beyond the culturally adapted lifestyle program which included structured sessions on diet, physical activity, and behavior change. The qualitative component explored participants’ perceptions within this program without independent outcome testing.
(Bennet et al., 2022)	Sweden / 2022	The intervention consisted of a four month culturally adapted lifestyle program including group counseling in Arabic, cooking classes with a professional chef, and motivational and economic support for lifestyle change. Participants achieved an average weight reduction of 2.5 kg and a BMI decrease of 0.5 kg per square meter.
(Rieger et al., 2021)	Norway / 2021	The study suggested group based health initiatives addressing cultural norms around food and socialization to encourage supportive discussions about health and weight among Turkish immigrant women. No intervention was implemented.

## 4 Discussion

### 4.1 Prevalence of overweight and obesity

This scoping review synthesized 20 studies examining the prevalence of overweight and obesity among immigrant adults in Nordic countries (Qureshi et al., 2020; Ahmed et al., 2018; Kjøllesdal et al., 2023 May; Persson et al., 2014; Kjøllesdal et al., 2023; Bennet et al., 2016; Skogberg et al., 2016; Gele et al., 2016; Carlsson et al., 2014; Tennakoon et al., 2013; Siddiqui et al., 2017; Qureshi et al., 2022; Iversen et al., 2013; Al-Majdoub et al., 2020; Kinnunen et al., 2018; Gele and Mbalilaki, 2013; Bennet et al., 2022; Sandstrom et al., 2015; Rieger et al., 2021; Olaya-Contreras et al., 2019). To our knowledge, this is the first scoping review to consolidate evidence on overweight and obesity among immigrant adults across all Nordic countries. Overall, the evidence consistently showed higher prevalence among immigrants than in general populations (Qureshi et al., 2020; Ahmed et al., 2018; Kjøllesdal et al., 2023 May; Bennet et al., 2016; Skogberg et al., 2016; Gele et al., 2016; Carlsson et al., 2014; Tennakoon et al., 2013; Siddiqui et al., 2017; Qureshi et al., 2022; Iversen et al., 2013; Al-Majdoub et al., 2020; Kinnunen et al., 2018; Gele and Mbalilaki, 2013; Bennet et al., 2022). The burden was particularly pronounced among Somali women and women from Middle Eastern backgrounds (Ahmed et al., 2018; Skogberg et al., 2016; Gele et al., 2016; Carlsson et al., 2014; Kinnunen et al., 2018). These findings align with broader European evidence, where female immigrants from the Middle East and North Africa exhibit higher obesity rates in France and Southern Europe (Toselli et al., 2014; Martin-Fernandez et al., 2012; Gautret et al., 2013; Gualdi-Russo et al., 2016).

Across the Nordic region, Somali, Iraqi, Kurdish, Turkish, Sri Lankan, and Russian immigrants were the most frequently studied populations, whereas evidence on newer or smaller groups such as Polish, Afghan, and Eritrean immigrants remains limited (Qureshi et al., 2020; Ahmed et al., 2018; Kjøllesdal et al., 2023 May; Bennet et al., 2016; Skogberg et al., 2016; Gele et al., 2016; Carlsson et al., 2014; Tennakoon et al., 2013; Siddiqui et al., 2017; Qureshi et al., 2022; Iversen et al., 2013; Al-Majdoub et al., 2020; Kinnunen et al., 2018; Gele and Mbalilaki, 2013; Bennet et al., 2022; Sandstrom et al., 2015; Rieger et al., 2021; Olaya-Contreras et al., 2019). This uneven representation may partly explain the variability in reported prevalence rates and emphasize the need for more inclusive research capturing diverse migrant communities. Notably, no eligible prevalence studies were identified from Denmark or Iceland, leaving significant geographic gaps in the regional evidence base.

In contrast to the Nordic pattern, a nationwide Portuguese study found a lower prevalence of overweight among immigrants (44.9 %) compared with natives (52.8 %), though the risk increased with longer residence, particularly among immigrants of African origin, illustrating the gradual loss of the Healthy Immigrant Effect over time (da Costa et al., 2017). This effect, described in other high-income countries, suggests that immigrants often arrive with a relative health advantage but gradually adopt less healthy behaviors as they acculturate (Murphy et al., 2017).

The observed variation across contexts likely reflects differences in migration histories, socioeconomic integration, and host-country environments. Nordic studies predominantly focused on long-term residents rather than newly arrived migrants, indicating that post-migration adaptation—characterized by dietary acculturation, changing physical-activity patterns, and environmental influences—plays a stronger role in shaping obesity outcomes than pre-migration health status. These findings suggest that post-migration adaptation processes, influenced by gender, cultural background, and duration of residence, play a critical role in shaping obesity outcomes among immigrants in the Nordic region.

### 4.2 Key determinants and risk factors for overweight and obesity

The determinants of overweight and obesity among immigrant adults in the Nordic region reflect an interaction of behavioral, biological, sociocultural, and structural influences that jointly shape health outcomes (Qureshi et al., 2020; Ahmed et al., 2018; Kjøllesdal et al., 2023 May; Persson et al., 2014; Kjøllesdal et al., 2023; Bennet et al., 2016; Skogberg et al., 2016; Gele et al., 2016; Carlsson et al., 2014; Tennakoon et al., 2013; Siddiqui et al., 2017; Qureshi et al., 2022; Iversen et al., 2013; Al-Majdoub et al., 2020; Kinnunen et al., 2018; Gele and Mbalilaki, 2013; Bennet et al., 2022; Sandstrom et al., 2015; Rieger et al., 2021; Olaya-Contreras et al., 2019). This synthesis provides the first consolidated overview of how multi-level determinants jointly influence obesity risk among immigrant adults in the Nordic region. The evidence highlights how individual lifestyle behaviors intersect with environmental and social constraints, producing patterns of excess weight that differ by gender, cultural background, and migration experience.

Individual-level determinants were consistently linked to physical inactivity, high-energy dietary patterns, and low health literacy (Qureshi et al., 2020; Ahmed et al., 2018; Bennet et al., 2016; Gele et al., 2016; Carlsson et al., 2014; Siddiqui et al., 2017; Qureshi et al., 2022; Al-Majdoub et al., 2020; Kinnunen et al., 2018; Gele and Mbalilaki, 2013; Bennet et al., 2022). Several studies identified metabolic risk factors such as insulin resistance, dyslipidemia, and hyperglycemia, particularly among Middle Eastern and African immigrants, reflecting the combined effects of post-migration dietary westernization and reduced physical activity (Bennet et al., 2016; Gele et al., 2016; Al-Majdoub et al., 2020). Gender-specific barriers were prominent: Somali, Kurdish, and Turkish women described difficulties engaging in exercise because of household responsibilities, religious restrictions, and the lack of female-only facilities (Persson et al., 2014; Siddiqui et al., 2017; Gele and Mbalilaki, 2013; Sandstrom et al., 2015; Rieger et al., 2021; Olaya-Contreras et al., 2019). Emotional eating during long, dark winters and social expectations of generosity at shared meals further contributed to weight gain (Rieger et al., 2021; Olaya-Contreras et al., 2019). Limited health literacy and low awareness of nutritional guidelines constrained the ability to adopt healthier diets (Kjøllesdal et al., 2023 May; Kjøllesdal et al., 2023; Iversen et al., 2013).

Structural and environmental determinants encompassed socioeconomic disadvantage, low educational attainment, limited employment opportunities, and restricted access to culturally adapted health information and preventive care (Qureshi et al., 2020; Skogberg et al., 2016; Tennakoon et al., 2013; Qureshi et al., 2022; Al-Majdoub et al., 2020; Kinnunen et al., 2018). Economic hardship and time pressure led to reliance on inexpensive, calorie-dense foods and reduced leisure-time physical activity (Qureshi et al., 2022; Kinnunen et al., 2018). Several studies noted that women with limited host-language proficiency or social networks faced isolation and disengagement from community health initiatives (Kjøllesdal et al., 2023 May; Kjøllesdal et al., 2023; Iversen et al., 2013; Rieger et al., 2021). These findings show how social and economic marginalization perpetuates obesogenic environments, particularly in urban immigrant communities.

Acculturation and duration of residence emerged as strong cross-cutting factors influencing obesity risk. Longer residence in the host country was generally associated with higher body-mass index, consistent with gradual lifestyle adaptation to sedentary urban settings and western dietary norms (Ahmed et al., 2018; Kjøllesdal et al., 2023 May; Gele et al., 2016; Gele and Mbalilaki, 2013). This pattern reflects the gradual erosion of the Healthy Immigrant Effect, whereby immigrants often arrive with better health indicators but experience declining outcomes as they adapt to new environments and behaviors (Murphy et al., 2017; Elshahat et al., 2022; Jen et al., 2015). The relationship between residence duration and obesity was particularly pronounced among women, who often faced reduced physical mobility due to lower workforce participation and family obligations. Conversely, limited language proficiency and low acculturation also predicted higher obesity rates, suggesting that incomplete social and cultural integration may reduce access to health information, preventive services, and social support (Kjøllesdal et al., 2023 May; Kjøllesdal et al., 2023; Iversen et al., 2013). These dual findings indicate that acculturation functions simultaneously as a behavioral and social pathway connecting migration to changes in weight.

Sociocultural influences played a central role in shaping diet and activity behaviors. Cultural expectations emphasizing food generosity, traditional high-calorie meals, and shared hospitality were reported as major contributors to overeating (Persson et al., 2014; Sandstrom et al., 2015; Rieger et al., 2021; Olaya-Contreras et al., 2019). At the same time, gender norms discouraging women from engaging in public exercise and climatic barriers, such as cold, dark winters, further restricted physical activity (Persson et al., 2014; Rieger et al., 2021; Olaya-Contreras et al., 2019). The persistence of these norms across multiple Nordic contexts indicates that cultural adaptation alone is insufficient without environments that enable equitable participation in health-promoting activities.

From a policy and environmental perspective, the reviewed studies linked obesity risk to broader socioeconomic and spatial inequalities, including neighborhood deprivation, limited walkability, and restricted access to affordable healthy foods (Qureshi et al., 2020; Kjøllesdal et al., 2023; Qureshi et al., 2022). However, few studies have explicitly examined how urban design, food systems, or public health infrastructure shape obesity among immigrants, indicating a major evidence gap in the Nordic literature. Comparative international evidence highlights that structural interventions, such as improving access to safe recreational spaces, subsidizing healthy foods, and integrating health promotion into language and integration programs, can address the “causes of the causes” of obesity (Ismail et al., 2022; Amstutz et al., 2020; Kumanyika, 2019; Leng et al., 2022; Fruh, 2017; Wadden et al., 2020).

Overall, the reviewed studies highlight that obesity risk among immigrant populations in the Nordic region is shaped by multi-level determinants encompassing individual behaviors, social integration, and structural factors.

### 4.3 Interventions to address overweight and obesity

The evidence on interventions addressing overweight and obesity among immigrant adults in Nordic countries is still limited. Existing trials, conducted primarily in Sweden, show that culturally tailored and gender-sensitive programs can produce measurable short-term improvements in weight and lifestyle behaviors (Siddiqui et al., 2017; Bennet et al., 2022). These studies involved Middle Eastern immigrants at high risk of type 2 diabetes and reported significant weight reduction and improved cardiometabolic outcomes. These findings indicate that intervention effectiveness was associated with cultural, linguistic, and behavioral adaptation in program design.

Beyond these tested approaches, several studies proposed context-specific strategies without formal evaluation. Recommended measures included combining health-literacy initiatives with language training to strengthen health knowledge and navigation skills (Kjøllesdal et al., 2023 May; Kjøllesdal et al., 2023; Iversen et al., 2013); using maternal and child-health services as outreach platforms for dietary and physical-activity counseling (Skogberg et al., 2016; Kinnunen et al., 2018); and creating safe, community-based environments for exercise, particularly for Somali women (Ahmed et al., 2018; Gele and Mbalilaki, 2013). Sweden’s national initiative, Physical Activity on Prescription (FaR), which links individualized exercise recommendations to subsidized access to fitness facilities, has been cited as a potentially transferable model for adaptation to immigrant populations, although its effectiveness for these groups has not yet been evaluated (Al-Majdoub et al., 2020).

International evidence—including programs from Switzerland and other European contexts—supports early, multi-level interventions that combine individual and population-based measures. Studies show that weight gain often accelerates in the early post-migration years, emphasizing the importance of preventive efforts soon after arrival (Mulugeta, 2020; Alidu and Grunfeld, 2018). Community programs offering cooking workshops, family-based physical activities, and culturally adapted nutrition education enhance participation and long-term sustainability (Amstutz et al., 2020). Structural measures that address the broader determinants of health, including food affordability, walkability, and urban design, complement individual-focused approaches and reduce socioeconomic barriers to healthy living (Kumanyika, 2019; Leng et al., 2022; Fruh, 2017; Wadden et al., 2020).

Overall, reviewed studies emphasized that effective approaches combined individual-level lifestyle counseling with structural and community-based components that broaden access to healthy behaviors. This review identifies a major evidence gap in tested interventions across Norway, Finland, Denmark, and Iceland, where culturally adapted programs remain largely unevaluated.

### 4.4 Potential impact of the study on public health policies and programs

This scoping review provides a comprehensive synthesis of evidence on overweight and obesity among immigrants in the Nordic region, highlighting both existing progress and persistent evidence gaps. This review consolidates obesity-related evidence across all Nordic countries and provides a comparative foundation for equitable policy development. The findings emphasize the importance of culturally responsive and evidence-informed public-health strategies that balance individual behavior change with broader structural and environmental supports. Integrating health literacy into language and resettlement programs, offering culturally tailored nutrition education, and expanding gender-sensitive physical-activity initiatives could enhance participation and effectiveness among diverse immigrant groups.

Although many determinants of obesity are well recognized internationally, this review adds region-specific insights by mapping how these factors manifest in Nordic welfare contexts. The evidence shows that socioeconomic inequality, linguistic barriers, and urban-design constraints interact with cultural norms to shape obesity risk among immigrants. Addressing these overlapping influences requires complementing individual-level initiatives with population-based measures that promote healthy food environments, equitable access to recreation spaces, and walkable urban planning.

The current literature remains uneven across countries and population groups. Somali and Middle-Eastern immigrants are relatively well studied, whereas evidence concerning newer or smaller communities—such as Polish, Afghan, and Eritrean migrants—is limited. Filling these gaps is crucial to ensure that preventive strategies and policy frameworks reflect the diversity of immigrant experiences and needs across the Nordic region.

Rather than proposing sweeping policy reforms, this review contributes a focused evidence base to guide incremental, contextually grounded improvements in immigrant health promotion. Findings from this review may inform Nordic health authorities seeking to strengthen inclusiveness and equity in obesity prevention efforts. These strategies align with ongoing Nordic efforts to promote participation, equity, and cultural competence in public-health and chronic-disease-prevention programs.

### 4.5 Suggestions for future research

Building on the evidence gaps identified in this review, future research should move beyond documenting prevalence to examine the mechanisms through which cultural, social, and structural factors jointly shape obesity among immigrant populations in the Nordic region. Studies should explore how acculturation trajectories, community environments, and welfare-system contexts influence dietary practices, physical activity, and metabolic outcomes over time. Particular attention is needed to understand how socioeconomic inequalities, neighborhood characteristics, and policy environments interact with individual behaviors to produce differential obesity risks across immigrant groups.

Longitudinal and mixed-method designs are essential to capture long-term health trajectories and to evaluate how structural and behavioral changes unfold after migration. Future trials should also identify which intervention components—such as language-integrated health education, culturally adapted dietary counseling, or gender-responsive physical-activity programs—are most effective in achieving sustainable outcomes and reducing inequities. Evaluating policy measures that promote healthy food environments, improve walkability, and enhance access to affordable recreational spaces will provide valuable evidence on upstream structural determinants—the “causes of the causes”—of obesity in immigrant populations.

Expanding research beyond the relatively well-studied Somali and Middle Eastern groups to include smaller and emerging communities, such as Polish, Afghan, and Eritrean immigrants, will strengthen the inclusiveness and representativeness of Nordic evidence. Expanding the evidence base across diverse immigrant groups will enable the development of equitable, culturally competent, and population-level obesity-prevention strategies aligned with Nordic public health priorities.

### 4.6 Strengths and limitations

This scoping review draws upon evidence from four major databases (CINAHL, PsycINFO, PubMed, and Web of Science) to provide an integrated understanding of overweight and obesity among immigrants in the Nordic countries. The inclusion of both quantitative and qualitative studies enabled a multidimensional understanding of behavioral, sociocultural, and contextual factors shaping obesity. Restricting the search to publications from 2013 to 2023 ensured that the synthesis reflected contemporary migration patterns, public-health priorities, and policy environments in the region. The review followed PRISMA-ScR and JBI methodological guidance, enhancing transparency and reproducibility across all stages of data collection, screening, and synthesis.

Several limitations should nevertheless be acknowledged. First, the reliance on a limited number of databases may have resulted in the omission of relevant studies not indexed in these sources, while the exclusion of gray literature—such as community reports, policy briefs, and local evaluations—may have overlooked practical insights from community-based interventions. Second, restricting the search to English-language publications, although facilitating consistency across databases, likely excluded valuable research published in Nordic or other regional languages, thereby narrowing the contextual scope of the findings. Third, while MeSH terms were considered during search development, the final strategy relied primarily on free-text keywords to ensure cross-database compatibility, which may have led to the omission of studies indexed under alternative controlled vocabulary terms. Fourth, consistent with scoping-review methodology, no formal critical appraisal of study quality was conducted, meaning that the findings should be interpreted descriptively rather than inferentially. Fifth, the geographic distribution of the included studies was uneven, with most originating from Norway and Sweden and limited representation from Denmark, Finland, and Iceland, constraining the regional generalizability of the conclusions. Finally, the absence of longitudinal and intervention data limits the ability to assess temporal dynamics or causal mechanisms in immigrant weight trajectories.

## 5 Conclusions

This scoping review synthesized current evidence on overweight and obesity among immigrant adults in the Nordic countries, outlining prevalence patterns, key determinants, and intervention approaches. The findings confirm that overweight and obesity are disproportionately common among immigrant populations and are influenced by interacting behavioral, socioeconomic, and cultural factors. While most available studies have focused on Somali and Middle Eastern groups, evidence remains limited for smaller and newer immigrant communities, highlighting the need for broader, comparative research. By consolidating fragmented evidence across multiple Nordic contexts, this review provides the first comprehensive regional synthesis of its kind and identifies persistent gaps related to underrepresented populations, structural determinants, and intervention evaluation. The results offer a foundation for developing inclusive, culturally responsive, and equity-oriented obesity prevention policies across the Nordic region.

## Funding

This research did not receive any specific grant from funding agencies in the public, commercial, or not-for-profit sectors.

## CRediT authorship contribution statement

**Luna Emilia Kronstad:** Writing – review & editing, Writing – original draft, Visualization, Validation, Software, Resources, Methodology, Formal analysis, Data curation, Conceptualization. **Miroslava Tokovska:** Writing – review & editing, Writing – original draft, Supervision, Methodology, Formal analysis, Conceptualization. **Adnan Kisa:** Writing – review & editing, Writing – original draft, Supervision, Methodology, Formal analysis, Conceptualization.

## Declaration of competing interest

The authors declare that there are no financial or personal relationships with other people or organizations that could inappropriately influence (bias) their work. Specifically, there are no potential competing interests, including:

• Employment

• Consultancies

• Stock ownership

• Honoraria

• Paid expert testimony

• Patent applications or registrations

• Grants or any other funding

All authors have completed the Declaration of Interests tool and have nothing to declare.
